# Short-term low salinity mitigates effects of oil and dispersant on juvenile eastern oysters: A laboratory experiment with implications for oil spill response activities

**DOI:** 10.1371/journal.pone.0203485

**Published:** 2018-09-07

**Authors:** Meagan Schrandt, Sean Powers, F. Scott Rikard, Wilawan Thongda, Eric Peatman

**Affiliations:** 1 Department of Marine Sciences, University of South Alabama, Mobile, Alabama, United States of America; 2 Dauphin Island Sea Lab, Dauphin Island, Alabama, United States of America; 3 Auburn University Shellfish Laboratory, Dauphin Island, Alabama, United States of America; 4 School of Fisheries, Aquaculture and Aquatic Sciences, Auburn University, Auburn, Alabama, United States of America; Xiamen University, CHINA

## Abstract

Following the Deepwater Horizon oil spill, eastern oyster (*Crassostrea virginica*) reefs in the northern Gulf of Mexico were exposed to oil and various associated clean-up activities that may have compromised oyster reef health. Included in the exposure was oil, dispersant, and in some locales, atypical salinity regimes. Oil and dispersants can be detrimental to oysters and the effects of salinity depend on the level. In addition to these extrinsic factors, genetic diversity of oyster populations may help the oysters respond to stressors, as demonstrated in other systems. We used a 3×3×2 factorial design to experimentally examine the effects of oil/dispersed oil, intraspecific genetic diversity, and salinity on juvenile (ca. 25 mm shell height) oyster survivorship and growth during a 21-d exposure in a closed, recirculating system. The genetic effect was weak overall, oil and dispersed oil negatively affected juvenile oyster survivorship, and low salinity mitigated mortality in oil and dispersed oil treatments. Survivorship was about 40% greater in low-salinity than in mesohaline water for both oil and dispersed oil treatments, bringing survivorship in low salinity oil-only treatments to a similar level with low salinity controls (no oil). Oyster growth was minimal after 21 d but appeared to be negatively affected by oil and dispersed oil, and had a significant interaction with salinity. Our results may be informative for future decisions regarding oil spill response activities and suggest that a pulse of low salinity water may be a viable short-term mitigation option for oysters if filtration characteristics, exposure time, and water temperatures are all considered, in addition to weighing the costs and benefits of this type of response on other organisms and habitats.

## Introduction

Vulnerability of nearshore habitats like eastern oyster (*Crassostrea virginica*) reefs, is important to resource managers because these habitats are valued worldwide for their ecosystem services. Successive reproduction and settlement on existing oyster reef structure results in an ecologically important system that provides food, shelter and habitat for other organisms, as well as improves water quality and stabilizes bottom habitats [1]. In addition to the ecological importance of oyster reef habitats, the eastern oyster is one of the most economically important shellfish species in the Gulf of Mexico (GOM) (e.g., [2]), supporting numerous coastal communities. Being at the interface of land and sea, these ecologic and economically important nearshore ecosystems are at risk of impacts originating from both land and sea. In the spring of 2010 (April 20^th^), an explosion on the Deepwater Horizon (DWH) drilling rig, located 66 km off the Louisiana coast, caused an oil and gas blowout at the BP-operated Macondo Prospect (MS252). Over the next 87 days, millions of barrels of oil entered offshore waters, and several million liters of the dispersants Corexit 9527 and 9500A^®^ were applied at the wellhead and on the ocean surface to disperse the slick [3]. Eventually, ocean currents, winds, and waves transported oil slicks toward the shoreline [4]. Louisiana was the first coastline contaminated by the oil, followed by Mississippi, Alabama, and Florida [5]. Coastal response to the DWH oil spill varied along coastlines to prevent oil contamination of nearshore habitats (e.g., saltmarsh, oyster reefs) generally located behind barrier islands. For example, Louisiana opened fresh water diversion structures to physically push contaminated waters from the coast, which resulted in an extended period of low salinity in estuarine waters during warm summer months [6]. In contrast, Alabama filled a breach along a barrier island, elevating salinity in Mississippi Sound [7–8]. Mechanical methods were also used to remove visible oil from estuarine habitats [9]. Therefore, nearshore ecosystems were likely subjected to multiple stressors including oil, dispersants, and atypical salinity regimes during the response period.

Petroleum hydrocarbon contaminants can have sub-lethal and lethal consequences for marine organisms. For crude oil, most toxic effects are attributed to aromatic compounds, specifically polycyclic aromatic hydrocarbons (PAHs). PAHs are often metabolized by marine organisms (e.g., [10]) and can bioaccumulate (e.g., [11]). During the DWH oil spill, coastal marine organisms were exposed to oil, and potentially dispersant, advected from offshore. Sammarco and colleagues [12] reported average PAH and TPH (total petroleum hydrocarbons) concentrations in northern GOM coastal waters from May through August 2010 of 0.047 ppm and 202.206 ppm, respectively. Wade and colleagues [13] reported that most water samples tested for PAH concentration in the BP Water Chemistry Data set (April through August) were classified as background concentrations, with elevated PAHs mostly within 25 km of the wellhead. The results were similar for TPH as most samples (84%) had concentrations that could be considered background, with elevated concentrations reported from areas near the leaking well or collected at the surface to characterize the released oil [13]. Although surface coastal waters were subjected to oiling, rapid decreases in petroleum hydrocarbon levels during sequential sampling from May 2010, August 2010, and May 2011 suggested that surface oil was rapidly weathered in nearshore coastal waters and/or diluted by physical processes [14]. In addition to the PAHs and TPHs, the use of dispersants as one response activity added another type of hydrocarbon to the mix of potential stressors. Dispersants are a proprietary mixture of surfactants and hydrocarbon-based solvents and although the dispersants used are considered by manufacturers to be non-toxic and biodegradable when used independently and at recommended concentrations, the combination of oil and dispersant may alter the toxic effects (e.g., [15]). Therefore, the extensive use of dispersant during the DWH oil spill in offshore waters has led to questions of whether dispersant should be utilized in future oil spills in coastal waters.

Another stressor that may have affected coastal ecosystems, specifically oyster reefs, was the alteration of natural salinity regimes. Optimal physiological salinities for eastern oysters are mesohaline, ranging from 10–20 ppt [16]. Lower salinities reduce larval growth and settlement [17] while higher salinities, in addition to creating physiological stress, can increase salinity-dependent predation by marine snails (e.g., [18]). Both reduced and elevated salinity regimes occurred during DWH response activities, with decreased salinities in certain coastal areas of Louisiana resulting from the opening of freshwater diversion structures to physically push contaminated water from coastlines [6] and increased salinities in Mississippi Sound in Alabama after filling a breach along a barrier island to protect the mainland shorelines [7–8]. The altered salinity regimes occurred during the warm summer months, already a time of high physiological stress due to the heat, and coincided with oyster spawning (mid-spring through late fall) [19, 16] when meroplankton larvae are developing in the water column and settling gregariously on subtidal and shoreline oyster reefs in the northern GOM. Hence, multiple life stages of oysters from pelagic larvae to benthic, sessile juveniles and adults may have been impacted by the various stressors associated with the oil spill and subsequent response activities.

Although there were likely multiple stressors imposed on northern GOM oyster reefs during the DWH oil spill and response, the reefs may have had mechanisms for coping with stress. One such mechanism is the potential for genetic diversity to ameliorate stress through effects on ecological processes. Genetic diversity within populations has been shown to affect productivity and fitness, growth, population stability, inter-specific interactions, and ecosystem-level processes [20]. For oysters specifically, increased genetic diversity can have positive effects for larval settlement on existing oyster reefs [21], as well as growth and recruitment in the absence of predators [22]. Underlying genetic variation in traits related to stress response and population dynamics of oyster populations may conceivably aid in resistance, and ultimately resilience, to ecological disturbances, but this has not been previously tested in oysters.

The potential ecological effects of the myriad of oil spill response activities have been debated, along with the role that natural biological characteristics or mechanisms play in resistance to disturbances. We used a mesocosm experiment to examine how intraspecific oyster diversity affects resistance to the combined effects of low salinity, oil, and dispersed oil. We experimentally manipulated juvenile oyster genetic diversity and subjected oysters in mesohaline and low-salinity conditions to a pulsed exposure to oil and oil+dispersant, measuring lethal (survivorship) and sub-lethal (growth) effects. Results from research on the combined effects of contaminants and associated clean-up activities can be used to inform management and response protocols in future spills.

## Materials and methods

Five pairs of adult eastern oysters were induced to spawn in May 2016 at the Auburn University Shellfish Laboratory located on Dauphin Island, Alabama. The ten adult oysters were selected from different breeding lines established at the laboratory to produce seed oysters of similar growth rates and appearances for commercial and research purposes. The offspring of each of the five pairs (labeled A–E) were kept separate and grown in an upwelling flow-through system to a seed size of approximately 25 mm shell height (SH; maximum distance from umbo to valve edge) before experimental use. The flow-through system pumped water in from the GOM, filtered it multiple times, and flowed through the upwelling tanks before being released to the GOM, so oysters were exposed to natural variations in temperature and salinity during their grow-out period (ca. 4–5 months). Genetic diversity in the experiment was manipulated by creating monocultures and polycultures of the different offspring groups, assuming genetic variation within offspring monocultures (full siblings) was less than that among offspring polycultures (multiple groups of full siblings). Genetic diversity was confirmed with an eastern oyster-specific 58-SNP (single nucleotide polymorphism) multiplex panel analysis, developed and tested with GOM oysters [23], followed by genetic STRUCTURE analysis. The STRUCTURE software package is used to investigate population structure from multi-locus genotype data, including inferring distinct populations from the sample and assigning individuals to populations. We used this analysis to confirm genetic differences among genetic treatment levels. To test the hypothesis that increasing intraspecific diversity affects resistance to multiple environmental stressors, we manipulated oyster genetic diversity in three levels: offspring monocultures (A, B, C, D, E), mixtures of offspring from two different randomly-selected parental pairs (AB, BD, AE), and mixtures of offspring from three different randomly-selected parental pairs (CDE, BCE, ABC).

To manipulate multiple stressors that nearshore oysters were exposed to during the DWH oil spill and clean-up activities, the eleven genetic mixtures were subjected to each of six water treatments: (1) mesohaline seawater, (2) low salinity seawater, (3) mesohaline seawater with oil, (4) low salinity seawater with oil, (5) mesohaline seawater with oil+dispersant, and (6) low salinity seawater with oil+dispersant. Each of the eleven genetic mixtures had five replicates per treatment. Salinity regimes were regulated to 15–20 ppt for mesohaline systems and 5–10 ppt for low salinity systems. Oil (surrogate MC252, requested through BP) was added to the systems at a concentration of 1 ppt (1 ml oil per 1 L water) to ensure oyster exposure after predicted losses resulting from adsorption to the experimental tanks and system plumbing. For oil+dispersant treatments, the dispersant SlickGone (Dasic International Ltd., substituted for Corexit due to difficulties in obtaining Corexit) was mixed with the oil at a ratio of 1:20 dispersant:oil, the manufacturer recommended ratio, prior to addition to the system. For oil and oil+dispersant additions, all quantities were measured in clean amber glass jars and added to the sump tank to be pumped up into each of the smaller experimental tanks (see below).

The experiment was conducted in September/October 2016 at the outdoor covered mesocosm facility at the Dauphin Island Sea Lab, Dauphin Island, Alabama. Six shelving systems were constructed (one for each of the six water treatments) and each system held 55 individual 5 L plastic aquaria (polycarbonate, food safe containers with internal dimensions: 26.5 cm L x 12 cm W x 14 cm H) distributed among three vertical shelves. A 416 L sump tank was located beneath and a magnetic drive pump (115V, 60 Hz, 3A; Pentair Aquatic Eco-Systems, Cary, NC) moved water from the sump to each of the shelves. Individual aquaria had separate valves and water lines and each could fill, overflow into a tray on the shelf, and drain into the sump, creating a re-circulating system. Water flow rates were monitored to maintain complete individual tank turnover in approximately 8–10 minutes. No filters were added to the system so as not to remove the oil and/or dispersant. Each system contained approximately 550 L of water. Water treatments were randomly assigned to systems.

After the six systems were constructed, seawater was added and circulated for 10 days before the addition of oysters. Water was pumped in from Mobile Bay, settled 7–10 d, filtered through a rapid sand filter followed by a UV filter, and diluted with dechlorinated fresh water via an activated carbon filter to a salinity of 18 ppt. Oysters (48 per aquaria, with 2- and 3-polycultures containing 24 and 16 oysters of each group, respectively; 15,840 total) were added on September 6, 2016 and a subset was measured for shell height (13–43 mm, mean = 24.9 mm). Genetic mixtures were randomly assigned to each tank within a shelf and to each shelf within a system. For 13 d, oysters were acclimated to the systems before the salinity was lowered with fresh water to 8 ppt in the three low salinity systems. After the addition of dechlorinated fresh water, all systems had the same water level and equilibrated overnight until salinities stabilized. The following morning, oil and oil+dispersant was added to the appropriate systems as a single pulsed addition. The experiment ran for 21 d. Temperature and salinity were regularly monitored and dechlorinated water (via aeration for 3+ d to bubble out the chlorine) was periodically (approximately every 2–3 d) added to replace evaporated water and maintain sufficient levels for pump function. Photographs of the systems were taken throughout the experiment to document changes in water clarity. Oysters were fed the mixed-algae concentrate Shellfish Diet 1800 (Reed Mariculture Inc., Campbell, CA, USA) twice daily at a rate of 5 mL algae concentrate per 100 g meat wet weight, per manufacturer recommendations. Average wet weight among a subset of oysters from each monoculture produced for the experiment but not used in experimental tanks was used to determine algae quantities. After 21 days, oysters were removed from the systems. Live and dead oysters were counted and a subset of surviving oysters was measured for shell height to assess growth. Percent survivorship (arcsine-transformed) and difference in shell height (mm) were assessed via 3-way ANOVA in JMP 11.0.

A time series of water samples was analyzed for PAH and TPH (total petroleum hydrocarbons). Water samples were collected from just below the water surface in the sump tank at 1 h, 24 h, 48 h, 3 d, 8 d, and 14 d after oiling. In addition to these samples, water was also collected from one tank on each of the three shelves at the 1 h and 24 h time periods from select systems, to ensure the movement of oiled water up through the entire system. All samples were collected in amber glass jars, placed on ice and shipped overnight for processing by an EPA-certified laboratory (Pace Laboratory, St. Rose, LA), using protocols 8270 MSSV and 8015M/ORO Organics for PAH and TPH, respectively.

## Results

### Ensuring treatment level differences

**Fig 1:**
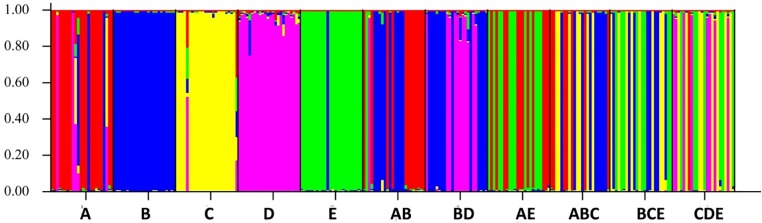
STRUCTURE analysis of genetic treatment levels. Bar plot resulting from STRUCTURE analysis based on an eastern oyster-specific SNP multiplex panel for a subset of oysters (24 oysters per genetic group) used in the mesocosm experiment. Offspring from the five parental pairs are labeled A–E and polycultures are random mixes of the monocultures. Each vertical line (bar) represents an individual oyster. For example, a solid blue vertical bar is an oyster with a genetic make-up from the “B” group, whereas an oyster with a vertical bar of two different colors indicates a genetic make-up from the two groups corresponding to the two colors.

We successfully varied genetic diversity by mixing offspring of the parental pairs. Results from the eastern oyster-specific SNP multiplex panel and subsequent STRUCTURE analysis clearly separated the five monocultures (A–E) and the polycultures into genetically distinct groups (Fig 1). Each polyculture was a genetic mix of the monocultures, where individuals could be matched back to their parental pair.

**Fig 2:**
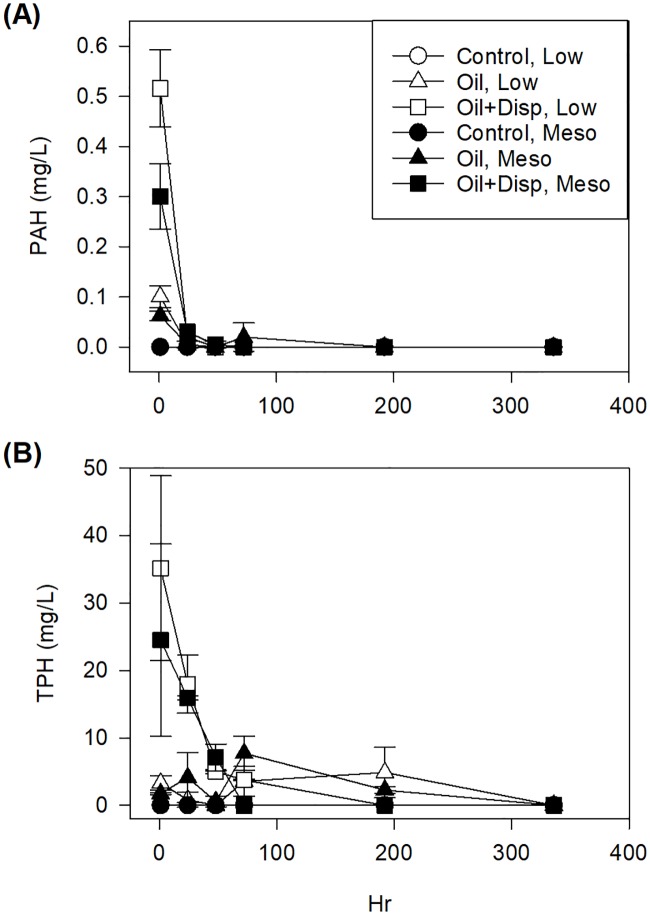
Water sample petroleum hydrocarbon levels. Time series analysis of water samples for (A) PAH and (B) TPH. Samples were collected 1 h, 24 h, 48 h, 3 d, 8 d, and 14 d after oiling the experimental systems.

The water sample time series documented a significant decrease in both PAH and TPH levels after 24 h (Fig 2). One hour after oiling, control systems recorded 0 mg/L PAH and TPH. Oiled systems had intermediate levels (0.06–0.1 mg/L PAH and 1.7–3.3 mg/L TPH) and oil+dispersant systems had highest levels (0.3–0.5 mg/L PAH and 24.5–35.1 mg/L TPH). After 24 h, PAH concentrations were <0.03 mg/L in all systems and TPH concentrations were <5 mg/L in oil systems and <18 mg/L in oil+dispersant systems. TPH levels decreased to <7 mg/L in oil+dispersant systems after 48 h and <5 mg/L after 3 d.

**Fig 3:**
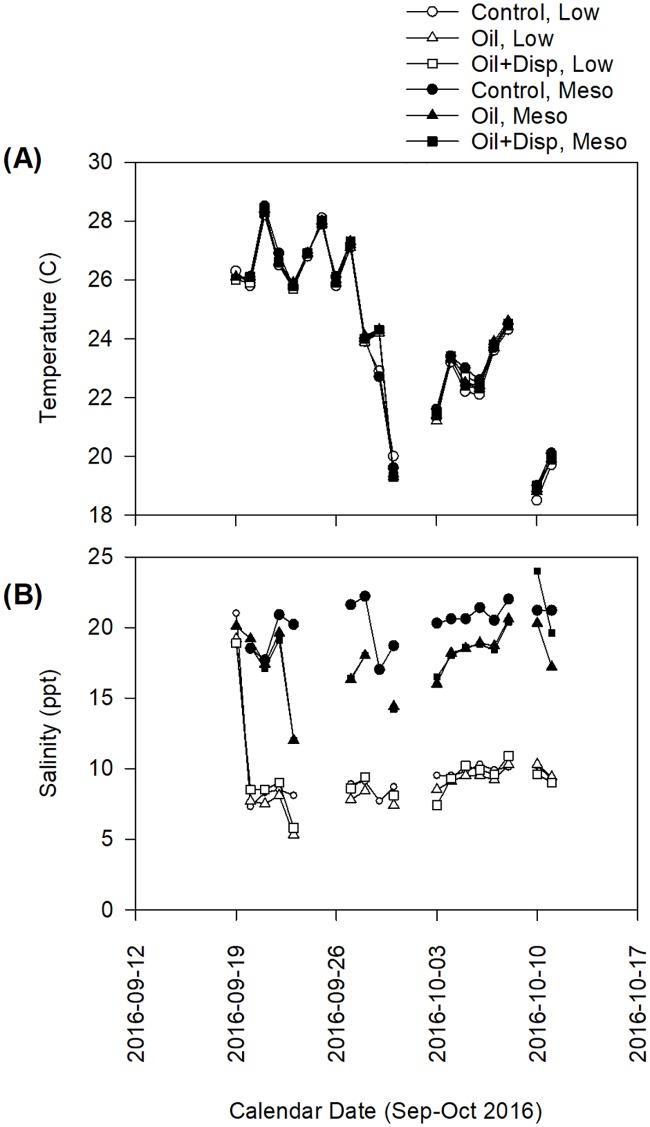
Temperature and salinity in experimental tanks. Time series analysis of (A) temperature and (B) salinity for the duration of the 21-d multi-stressor mesocosm experiment. Dates are formatted as year-month-day.

Temperature varied similarly over time in all six systems, regardless of oil or salinity treatments (Fig 3A). Two cold-fronts occurred and were reflected in decreased tank temperatures on September 28 and October 10, 2017. Salinity ranged from 17–21 ppt in all systems before the application of treatments. On September 20, 2017, the decrease in salinity for all low-salinity treatments is visible (Fig 3B). Mesohaline salinities generally did not fall below 16 ppt. Low salinity treatments typically remained between 8 and 10 ppt. Although all systems had some variation in salinity, the three low salinity treatments remained different from the three mesohaline treatments throughout the 21-d experiment.

### Visual observations

Consistent monitoring and photographing of all systems revealed visible differences among treatments. Within three minutes of oil addition, an oil slick was present on the surface of the sump tanks, but the water below remained clear. In oil+dispersant sumps, the whole water column within the sump tank turned a cloudy, rust-brown color and the slick was not as apparent. Within 10 minutes, this water was observed entering individual tanks. After three days, oiled sumps had brown-colored foam present on the surface but relatively clear water below. Oil+dispersant sumps had a less dense-looking foam than oiled sumps, but the water below remained cloudy, rust-brown.

**Fig 4:**
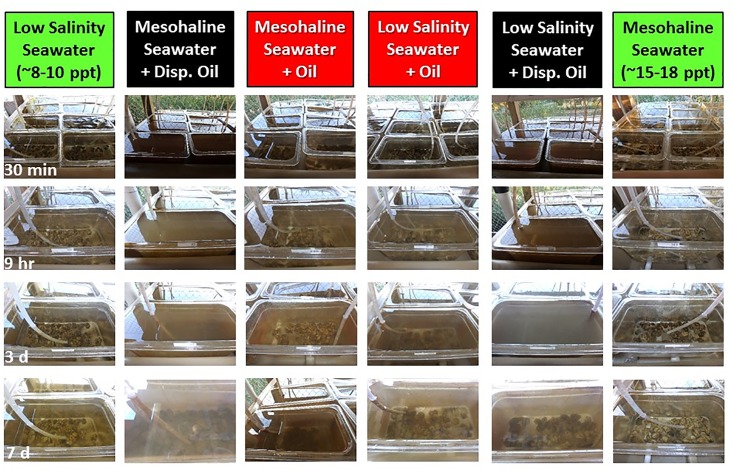
Changes to water clarity with oil and oil+dispersant additions. Photographic representation of visual observations for each treatment system during the experiment. Photos are provided for 30 min, 9 h, 3 d and 7 d after oiling. All photographs are from individual tanks on the bottom shelf of the unit (closest to the sump tank and pump). Treatments are color-coded along the top row of the figure and the time series can be followed down each column.

Time series photographs are presented for each system (including controls) for 30 min, 9 h, 3 d, and 7 d after treatment application (Fig 4). All photos were taken from the bottom shelf of the shelving systems. Water in control systems remained clear throughout the experiment. Water in oil systems appeared clear but had an oil sheen on the surface until approximately 3 d, when tanks became cloudier and the sheen remained. Water in oil+dispersant systems was brown and the oysters could not be seen until day 7 when the water began to clear.

### Oyster survivorship and growth

**Fig 5:**
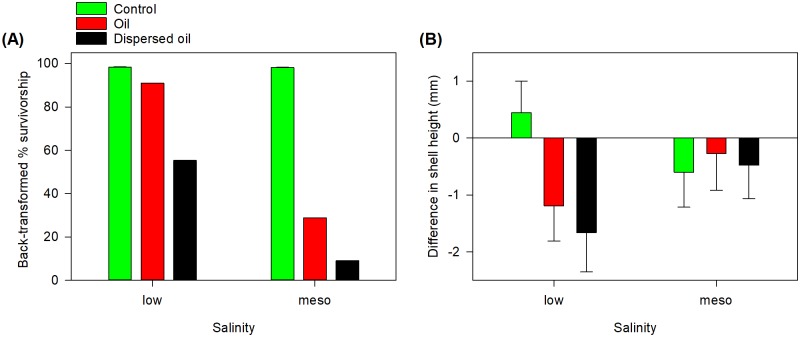
Salinity and oil effects on juvenile oysters. (A) Survivorship and (B) growth of juvenile oysters (mean ± SE) subjected to various salinity, oil, and genetic treatments for 21-d.

**Fig 6:**
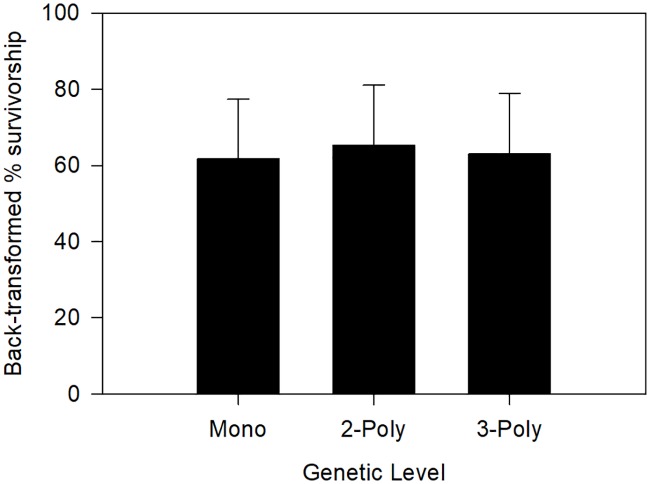
Effects of genetic diversity on juvenile oyster survivorship.

All three main factors, as well as the oil × salinity interaction, significantly affected oyster survivorship (Table 1). Oil, oil+dispersant, and mesohaline salinity in the non-control treatments decreased oyster survivorship, regardless of genetic diversity (Fig 5A). Control systems had 95.5 ± 0.5% survivorship at low salinity and 98.2 ± 0.3% survivorship in mesohaline conditions. In oil systems, back-transformed survivorship was 94.9 ± 1.0% in low salinity water and 28.7 ± 0.07% in mesohaline water. Oysters exposed to oil+dispersant had survivorship of 55.3 ± 0.12% in low salinity and 9.1 ± 0.08% survivorship in mesohaline conditions. The genetic effect was weaker (Table 1), with greatest survivorship in the 2-polyculture oysters (Fig 6).

**Table 1:** Effects of genetic diversity, oil and salinity on juvenile eastern oyster (3-way ANOVA).

		Survivorship		Growth	
Source	DF	F	P	F	P
Genetic Level	2	8.449	**0.0003**	0.785	0.4562
Salinity	1	834.140	**<0.0001**	1.473	0.2251
Oil	2	1230.026	**<0.0001**	4.006	**0.0184**
Genetic Level × Salinity	2	2.149	0.1184	1.352	0.2589
Genetic Level × Oil	4	0.592	0.6690	0.512	0.7269
Salinity × Oil	2	203.290	**<0.0001**	5.916	**0.0027**
Diversity Level × Salinity × Oil	4	0.349	0.8477	1.009	0.4013

Three-way ANOVA results for the effects of genetic diversity, salinity and oil on juvenile *Crassostrea virginica* survivorship and growth (difference in shell height) after the 21-d closed, recirculating, mesocosm experiment.

Oyster growth, as measured by the difference in shell height of a subset of surviving oysters, was affected by oil treatment and the oil × salinity interaction (Table 1). Most growth was apparently negative, which we attribute to the measurement of a subset of live oysters at the end of the experiment; we believe larger oysters suffered greater mortality so the subset measured at the end of the experiment would be smaller because of the measurement protocol. Growth was most negatively affected by oil+dispersant and then oil-only treatments (Fig 5B). Generally, growth was better in mesohaline as opposed to low salinity waters. Overall, growth was minimal, typically less than a millimeter during the 21 days.

## Discussion

We simulated a pulsed oil disturbance to nearshore/coastline oyster beds using a closed mesocosm system and tested the effects of multiple stressors on juvenile oyster survivorship and growth. A single pulse followed by re-circulation was used because shoreline reefs affected by the DWH oil spill likely experienced weathered oil by the time the oil was advected inshore; the outdoor recirculating system weathered and mixed the oil in our experiment. Although a seemingly high (1 ppt) oil concentration was initially applied to the sump tanks, PAH and TPH concentrations were generally less than those reported for northern GOM coastal waters during the DWH oil spill (e.g., [12–13]), especially after 48 h. Indeed, PAH concentrations were <0.03 mg/L within 48 h and TPH concentrations were <18 mg/L. These PAH concentrations are well below what is thought to produce adverse biological effects (i.e., ≥4022 ng/g). Thus, our results are conservative regarding contaminant concentrations despite an initially high dosage. It is important to note that the recirculating system is devoid of coastal processes like tides and currents that may aid in transport of contaminants away from oysters and dissipation of effects in natural shoreline habitats, but these processes could also act to re-contaminate reefs. The quick decline in PAH and TPH concentrations reflects reports by Liu and colleagues [14] of the rapid reduction in hydrocarbon concentrations after sequential sampling of coastal surface waters in the northern GOM from May to August 2010. Similar to other reported coastal conditions during the oil spill was the transient visual disturbance; water in oiled treatments remained relatively clear and oil+dispersant tanks cleared after 7 d. Along coastlines following the spill, varying degrees of oiling were observed, with some areas having re-occurring observations even after clean-up activities (reviewed by [4]).

Our results document a negative effect of oil and oil+dispersant on juvenile oyster survivorship, but the effects were mediated by low salinity. Juvenile oysters in mesohaline conditions that were exposed to oil or oil+dispersant for 21 d experienced mortality rates greater than 50%. Oysters in low salinity water, however, had less than 10% mortality in oiled treatments and less than 50% mortality in oil+dispersant treatments (Fig 5A). Typically, toxicity is tested by measuring early larval growth, survival, and morphological abnormalities in marine organisms as early life stages are generally more sensitive than adult stages ([e.g., [24–26]). In this study, we document mortality of juvenile oysters, which have already surpassed the larval and settlement stages, indicating that later life stages are also susceptible to adverse effects in a closed system. Previous experimental work has documented negative effects of oil exposure for oyster gametes [27–28]. Contrastingly, a study of the effects of chronic exposure of eastern oyster larvae to water-accommodated fractions of fresh and weathered oils from the DWH oil spill suggests that it is unlikely that there were significant negative effects on the growth and settlement of larvae [29]. Furthermore, Carmichael and coworkers [30] and Xia and colleagues [31] reported no significant carbon uptake by sub-adult and adult oysters during the DWH oil spill. The lack of adverse effects in the field may be due to the open system—coastal processes likely transport contaminated and non-contaminated waters to/from oyster beds, resulting in transient contamination and the potential for oysters to recover in non-contaminated waters. Alternatively, neither study provided direct evidence of exposure to oil so it may be possible that the oysters were not exposed to contaminants associated with the oil spill. Our results clearly demonstrate effects resulting from exposure in our closed loop system.

Aquatic organisms in the northern GOM were likely exposed to both oil and dispersant in combination, which may alter the toxic effects (e.g., [15]), and relatively little is known about the combined effects of oil and dispersant in nearshore environments [32]. Dispersed oil in our experiment always resulted in the lowest survivorship of oysters, regardless of treatment combination. Dispersants reduce interfacial tension at the oil-water interface, thereby facilitating mixing of oil into the water [33], which was visually observed in our experiment. The use of dispersants can reduce toxicity concentrations below thresholds for many marine species [34–35] though, and make oil slicks more accessible to hydrocarbon-degrading bacteria [36]. Nonetheless, various dispersants have been shown to negatively affect eastern oyster gametes and larvae [26–28] both alone, and when added to oil. By mixing oil into the water via the use of dispersant in our experiment, the oysters could readily uptake both oil and dispersant, potentially causing the observed mortality (although oyster tissue analysis was not conducted so we cannot confirm significant uptake of hydrocarbon contaminants). During the DWH oil spill, dispersant was used at the wellhead and at the surface [3] but was not applied in state waters. If dispersant and/or oil+dispersant did not reach the shorelines, oil may not have been as readily mixed within the water, decreasing the potential of filter-feeding organisms for contaminant uptake. Indeed, Fry and Anderson [37] reported minimal incorporation of DWH oil by barnacles and mussels and Carmichael et al. [30] and Xia et al. [31] reported no significant oil carbon uptake by oysters in DWH oil-affected areas in the northern GOM. We included the use of dispersant in our experiment though, because dispersed oil may have been transported to estuarine waters and our results could be used to inform management decisions and future response activities: the addition of dispersant to crude oil led to greater oyster mortality than oil alone. Further, debate on the use of dispersants in coastal (state) waters should evaluate the potential response action in the context of our results that demonstrated higher mortality of oysters in dispersed oil systems with limited water exchange.

In contrast to our prediction of low salinity being an additional stressor to nearshore oysters, low salinity lessened the negative effect on juvenile oyster mortality when oil and oil+dispersant were present. Survivorship was 95% in oil and 55% in oil+dispersant treatments at low salinity, which was greater than either oiling treatment in mesohaline salinity. We hypothesize oyster survivorship was greater in low salinity treatments with oil and oil+dispersant because physiological processes, like feeding (i.e., filtration) were depressed at low salinities and oysters likely closed their valves. A decrease in filtration would lead to less uptake of oil and oil+dispersant, also coinciding with the results reported by Fry and Anderson [37], Carmichael et al. [30], and Xia et al. [31] noting the lack of evidence of oil uptake by filter feeders during the DWH spill. This was a short-term (21-d) experiment though, and prolonged exposure to low salinities can be detrimental to oysters, regardless of the presence or absence of oil and dispersants. For example, prolonged low salinity (<5) during warm summer water temperatures negatively affected seed- and market-size oyster growth and survival in Breton Sound, Louisiana in 2010, an area affected by freshwater diversion [38]. The same study also reported that low salinity over a shorter period and not during warm (>25°C) temperatures had minimal effect on oyster growth or mortality. Our results suggest that a pulse of low-salinity may be a viable option for oysters for a short-term mitigation in the event of an oil spill or other similar disturbance, if (1) filtration uptake is the main mechanism for negative effects on oysters, (2) the length of exposure is not so long as to lead to oyster death via starvation or other mechanisms, and (3) the water temperature is relatively lower than the warm summer temperatures. As with all mitigation decisions, we advise consideration of the costs and benefits of the effects of freshwater flow for other organisms and habitats.

In addition to human responses to disturbance events, marine organisms likely possess characteristics and/or mechanisms to improve resistance to disturbance. For example, oysters have a broad salinity tolerance (5–40 ppt; [39]) and have been shown to have variation in physiology among populations and regions along the Atlantic coast [40–43]. These traits, which may be the result of underlying genetic variation, may aid in resistance, and ultimately resilience, to ecological disturbances. In fact, decreased genetic diversity can reduce species’ ability to adapt to environmental changes (reviews by: [44–45]). When assessing the relationship between biodiversity and response to a stressor, intraspecific diversity can be considered in a similar framework to that of interspecific diversity, which has been the typical measure of biodiversity in biodiversity-disturbance studies. For example, increased genetic diversity of eelgrass can mitigate effects of grazing [46] and heat stress [47]. Similarly, increased intraspecific diversity has been experimentally shown to increase settling success of barnacles [48]. In eastern oysters, genetic relatedness and cohort diversity positively affect growth and recruitment, respectively [22]. The responses in these examples can ultimately affect survivorship of the organisms. In our experiment, increased genetic diversity led to higher juvenile oyster survivorship overall, but the effect was weak compared to the oil and salinity factors. Greatest survivorship was in the two-polyculture treatment, not the three. This may be because the polyculture treatments were random mixtures of the monocultures, and certain monocultures could have been relatively more or less susceptible to the stressors in the experiment. For example, monoculture “D” oysters were typically larger than the others, meaning they may have needed to filter more water, thereby filtering more oil and dispersant, leading to relatively greater mortality of those oysters. The weak genetic effect overall may be reflective of natural eastern oyster response in the northern GOM as multiple studies have reported genetic similarities (i.e., low levels of population differentiation) throughout most of the GOM (e.g., [49–51].

At the end of the 21-d experiment, we also assessed oyster growth, wherein oiling and the interaction between oil and salinity both affected shell growth. With the short-term experiment, we were uncertain of the amount of growth to expect, and indeed, growth was rather minimal. Control oysters appeared to grow 1 mm in low salinity and either decrease by 1 mm or remain unchanged in mesohaline conditions. The apparent negative growth in any treatment can be explained by measuring only a subset of live oysters at the end of the experiment. If many of the dead oysters were relatively large compared to their living counterparts, the random subset measured after the experiment may have overall been smaller than the subset measured at the beginning. Nonetheless, the addition of oil and oil+dispersant both negatively affected oyster growth compared to control systems. In contrast to the oyster survivorship results, low salinity did not mitigate the effects on oyster growth. Instead, oysters in mesohaline salinities appeared to have less negative growth, as would be expected with oysters’ physiological preference for mesohaline salinity [16].

## Conclusions and management implications

We experimentally tested the effects of intraspecific genetic diversity and multiple stressors associated with an oil spill and its response activities on juvenile oyster survival and growth. The timing of the DWH oil spill coincided with spawning, settlement and peak growth season of GOM oysters, potentially subjecting nearshore oysters to oil, dispersant and low salinity water during multiple life stages. Results revealed, in the absence of predators and [presumably] disease, a negative effect of oil and oil+dispersant on juvenile oyster survivorship but positive effects of decreased salinity and increased genetic diversity. Growth was also affected by oil and oil+dispersant, and this effect was dependent on salinity, with oysters in mesohaline salinities experiencing a less-negative effect. Taken together, our results suggest that promoting and maintaining oyster genetic diversity may buffer environmental disturbance and/or change. Furthermore, low salinity (<10 ppt) seems to mitigate some negative effects of oil and oil+dispersant, which has important management and response-decision implications. Pulsed freshwater flow may temporarily protect oysters from uptake of contaminants, protecting oyster recruitment and resources in the affected areas.
